# A Mouse Model for Imprinting of the Human Retinoblastoma Gene

**DOI:** 10.1371/journal.pone.0134672

**Published:** 2015-08-14

**Authors:** Vasiliki Tasiou, Michaela Hiber, Laura Steenpass

**Affiliations:** Institute of Human Genetics, University Hospital Essen, University of Duisburg-Essen, Essen, Germany; University of Bonn, Institut of experimental hematology and transfusion medicine, GERMANY

## Abstract

The human *RB1* gene is imprinted due to integration of the *PPP1R26P1* pseudogene into intron 2. *PPP1R26P1* harbors the gametic differentially methylated region of the *RB1* gene, CpG85, which is methylated in the female germ line. The paternally unmethylated CpG85 acts as promoter for the alternative transcript 2B of *RB1*, which interferes with expression of full-length *RB1* in cis. In mice, *PPP1R26P1* is not present in the *Rb1* gene and *Rb1* is not imprinted. Assuming that the mechanisms responsible for genomic imprinting are conserved, we investigated if imprinting of mouse *Rb1* can be induced by transferring human *PPP1R26P1* into mouse *Rb1*. We generated humanized *Rb1_PPP1R26P1* knock-in mice that pass human *PPP1R26P1* through the mouse germ line. We found that the function of unmethylated CpG85 as promoter for an alternative *Rb1* transcript and as cis-repressor of the main *Rb1* transcript is maintained in mouse tissues. However, CpG85 is not recognized as a gametic differentially methylated region in the mouse germ line. DNA methylation at CpG85 is acquired only in tissues of neuroectodermal origin, independent of parental transmission of *PPP1R26P1*. Absence of CpG85 methylation in oocytes and sperm implies a failure of imprint methylation establishment in the germ line. Our results indicate that site-specific integration of a proven human gametic differentially methylated region is not sufficient for acquisition of DNA methylation in the mouse germ line, even if promoter function of the element is maintained. This suggests a considerable dependency of DNA methylation induction on the surrounding sequence. However, our model is suited to determine the cellular function of the alternative *Rb1* transcript.

## Introduction

Genomic imprinting is an epigenetic process leading to monoallelic gene expression, dependent on parental origin. The expression of imprinted genes is regulated by a gametic differentially methylated region (gDMR). gDMRs acquire methylation in only one of the parental germ lines, resulting in parental allele-specific methylation in the offspring. Regulation of imprinted gene expression by a gDMR is cis-acting, not gene-specific and applies to protein-coding genes and non-coding RNA genes organized in complex imprinted gene clusters [1, 2]. Using genome-wide analysis methods to determine either allele-specific gene expression or differential DNA methylation, about 150 imprinted genes have been identified in mice to date [3–7]. About half of these are conserved in humans and predominantly organized in gene clusters (updated lists can be found at www.otago.ac.nz/IGC for human and www.mousebook.org for mouse genes) [2].

In the genome-wide studies, single imprinted genes, which are not part of an imprinted gene cluster, have also been identified. Several of these are imprinted retrogenes. Retrogenes are defined as protein-coding genes that arose during evolution by retrotransposition of either transcribed mRNAs or transposable elements into introns of pre-existing genes or intergenic regions [8, 9]. Promoters of imprinted retrogenes are associated with CpG islands that are differentially methylated in the germ line. For example, the mouse imprinted retrogenes *Nap1l5*, *Mcts2*, *U2af1-rs1* and *Inpp5f_v2*, show expression restricted to the paternal allele due to a methylated gDMR in the promotor region of the maternal allele [10–13]. Interestingly, except for *U2af1-rs1*, these retrogenes also lead to imprinted gene expression of their host genes [13]. In contrast to retrogenes, pseudogenes are non-functional copies of genes that have lost their protein-coding potential. Pseudogenes also get integrated into intergenic regions or introns of genes by retrotransposition of spliced mRNAs. In humans, imprinting of the *RB1* gene is caused by insertion of the pseudogene *PPP1R26P1* into intron 2. So far, the *RB1* locus is unique for this type of imprinting control, representing a new subclass of imprinted genes. Imprinting of the *RB1* gene is not conserved in the mouse, as integration of *PPP1R26P1* did not occur until primate evolution [14, 15]. The mouse *Rb1* gene does not contain the *PPP1R26P1* pseudogene and does not show skewed imprinted expression of *Rb1* [16].

The *PPP1R26P1* pseudogene derives from the original gene copy of *PPP1R26* on chromosome 9 and was inserted in inverted direction in respect to the transcription direction of *RB1*. *PPP1R26P1* does not contain a functional open reading frame and promoter. Four additional copies of the *PPP1R26* original gene copy are found on human chromosome 22, but their location is intergenic instead of intragenic [14]. In *PPP1R26P1*, four small existing CpG-rich regions evolved into two larger CpG islands, CpG42 and CpG85. CpG42 acquires full DNA methylation on both parental alleles. CpG85 becomes methylated in the maternal germ line only and serves as gDMR of the *RB1* gene. In addition, CpG85 developed promoter activity, driving expression of an alternative, shortened *RB1* transcript, which is supposed to regulate expression of the full-length *RB1* transcript, possibly by transcriptional interference [16]. This is consistent with findings that maternally methylated DMRs are often found at intergenic positions and often act as promoters (reviewed in [17, 18]). Imprinting of *RB1*, therefore, fits in the concept stating that the emergence of new CpG islands during evolution provides the basis for genomic imprinting [19]. At *RB1*, the DNA sequence introduced by retrotransposition served as template for the evolution of new CpG islands, of which one developed into a differentially methylated gDMR.

Mouse *Rb1* is not imprinted because the *PPP1R26P1* pseudogene that provides the CpG island for gDMR function is not present. Since many imprinted loci are conserved from mice to men, we assume that the underlying mechanisms of imprinting are conserved in both species. We used the *Rb1* locus as template to experimentally test the consequences of pseudogene integration in respect to somatic and gametic DNA methylation and transcriptional regulation of the host gene. In vitro analysis of mouse embryonic stem cells (mESCs) carrying human *PPP1R26P1* showed that it also acts as cis-repressive element in mouse, resulting in skewed *Rb1* expression in favor of the allele not carrying the integrated pseudogene [20]. As imprint acquisition occurs in the germ line, we generated a humanized mouse model carrying a knock-in of human *PPP1R26P1* in intron 2 of *Rb1*. Here, we describe our in vivo studies on passaging the human *PPP1R26P1* pseudogene through the mouse germ line. DNA methylation studies show acquisition of DNA methylation on CpG85 in neuroectodermal tissues only, independent of parental transmission. Using expression studies, we show that promoter function of CpG85 is maintained in the mouse, resulting in expression of an alternative *Rb1* transcript, lacking exon 1 and exon 2. Expression of the alternative transcript results in repression of full-length *Rb1* transcription in cis. Our studies indicate that the function of *PPP1R26P1* as cis-repressive element of *Rb1* transcription is maintained but not controlled by imprinted differential DNA methylation.

## Results

**Fig 1:**
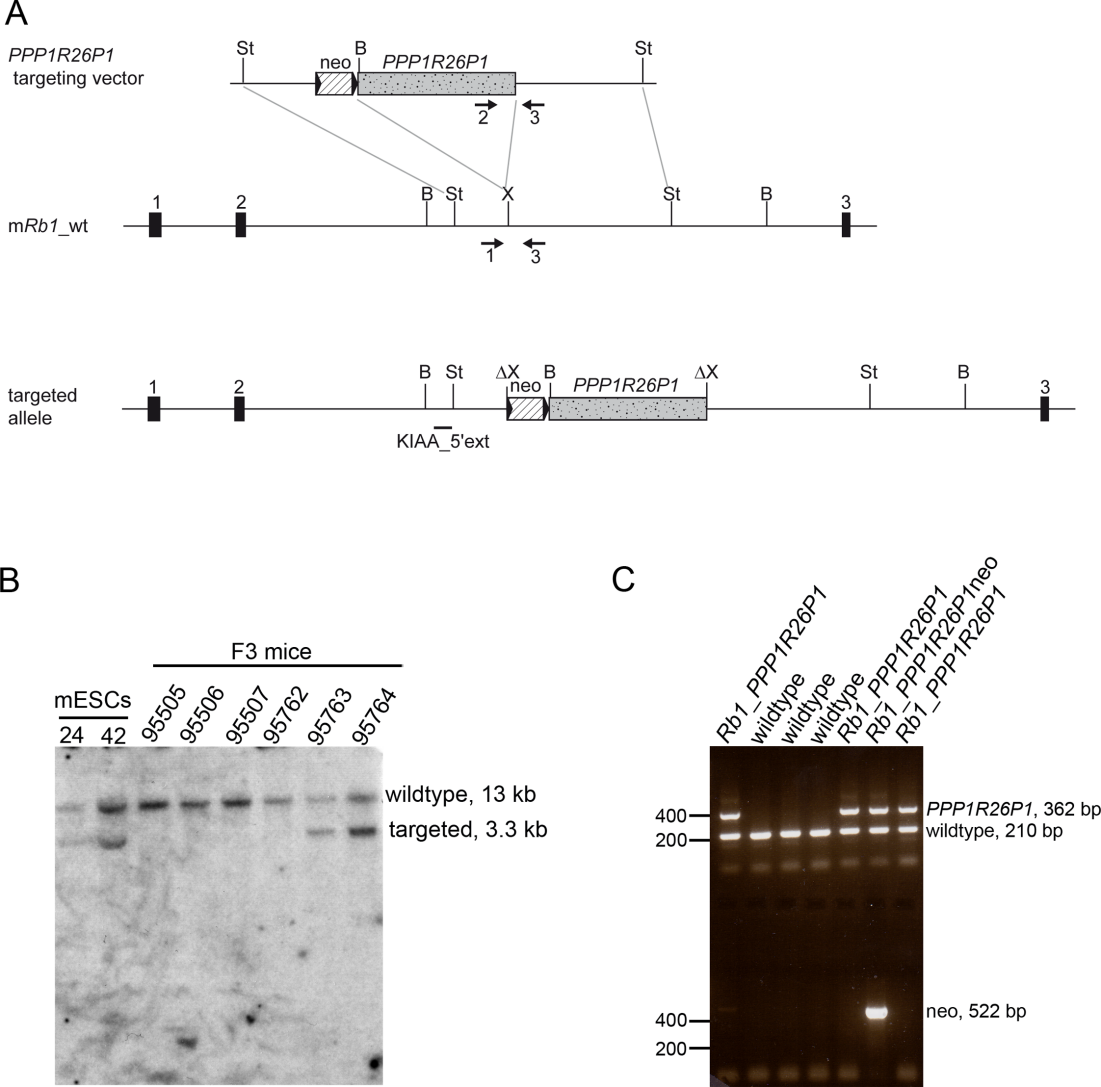
Generation of *Rb1_PPP1R26P1* knock-in mice. **A**) Top: Scheme of the targeting vector, consisting of two homology arms, a neomycin selection cassette flanked by loxP511 sites, and the *PPP1R26P1* human pseudogene. Middle: the wildtype mouse *Rb1* allele, with the site of integration shown. Bottom: the targeted and recombined allele. **B**) Southern blot of selected ES clones and tail snips of generation N1 *Rb1*_*PPP1R26P1*neo mice. Genomic DNA was digested with BamHI and hybridized with probe KIAA_5’ext, resulting in a 13 kb wildtype fragment and a 3.3 kb fragment of the targeted allele. **C**) Genotyping of generation N2 mice by PCR. To test for presence of *PPP1R26P1* and wildtype alleles, a three-primer PCR was used (primers 1, 2 and 3, indicated by arrows in A), yielding a 210 bp product for the wildtype allele (primers 1 and 3) and a 362 bp product for the *PPP1R26P1* allele (primers 2 and 3). A separate PCR was used for detection of the neomycin selection cassette, resulting in a 522 bp product for the cassette, if present. Black boxes: exons of the *Rb1* gene, striped box: neomycin selection cassette, stippled grey box: *PPP1R26P1*, black triangles: loxP511 sites; black horizontal line: Southern blot probe; arrows: PCR primer, indicating direction; restriction sites: St: StuI, B: BamHI, X: XhoI.

Human *PPP1R26P1* was integrated into intron 2 of the *Rb1* gene by homologous recombination in R1 mouse ES cells of strain 129 (Fig 1A). Construction of the targeting vector was described in previous work, which identified *PPP1R26P1* as a cis-repressive element [20]. The 5.2 kb *PPP1R26P1* fragment comprised the complete pseudogene as it is present in the human *RB1* intron 2. Successful homologous recombination was screened for by Southern blot and identified by an additional band of 3.3 kb in addition to the wildtype fragment of 13 kb (Fig 1B, left). In total, 24 of 169 clones were positive for correct integration of *PPP1R26P1* into the *Rb1* gene, corresponding to a targeting efficiency of 14%. Eight clones were chosen for karyotyping and four of them had a correct chromosome number of 40 (S1 Table). ES cells of clones 24 and 42 were chosen for injection into a total of 119 blastocysts of mouse strain C57BL/6J for the generation of knock-in mice. Seven chimeras from ES cell clone 24 exhibited only a low grade of chimerism and breeding of these chimeras did not result in germ line transmission of the modified *Rb1* allele. Seventeen chimeras obtained from ES cell clone 42 showed a high grade of chimerism and transmission of the *PPP1R26P1* allele through the germ line was successful. Germ line transmission was judged by agouti coat color of the animals and verified by Southern blotting digested genomic DNA isolated from tail snips (Fig 1B, right). The neomycin selection cassette, flanked by loxP511 sites, was removed by mating the generation N1 *Rb1*_*PPP1R26P1*neo mice with a CMV-Cre deleter strain of strain background C57BL/6J (for complete breeding scheme see S1 Fig). CMV-Cre deleter mice carry the transgene Tg(CMV-Cre)1Cgn on the X chromosome (females are heterozygous), which is expressed in all types of cells, including germ cells and the zygote [21]. Loss of the selection cassette in the N2 generation was screened for by PCR of genomic DNA (Fig 1C). Subsequent breeding was conducted to remove the Tg(CMV-Cre)1Cgn allele and to backcross *Rb1*_*PPP1R26P1* mice onto a C57BL/6J background. Screening of selected animals for presence of two microsatellite markers per chromosome showed pure C57BL/6J background in generation N5. *Rb1_PPP1R26P1* mice did not show any phenotype, weight, size and behavior were as for wildtype litter mates. Litter size was normal and genotype distribution was observed at mendelian ratios. Animals in generations N4 and N5 were used for analysis of DNA methylation at *PPP1R26P1*, expression of the alternative *Rb1* transcript 2B and skewing of *Rb1* expression.

### Human *PPP1R26P1* shows tissue-specific but not parent-of-origin-dependent DNA methylation

**Fig 2:**
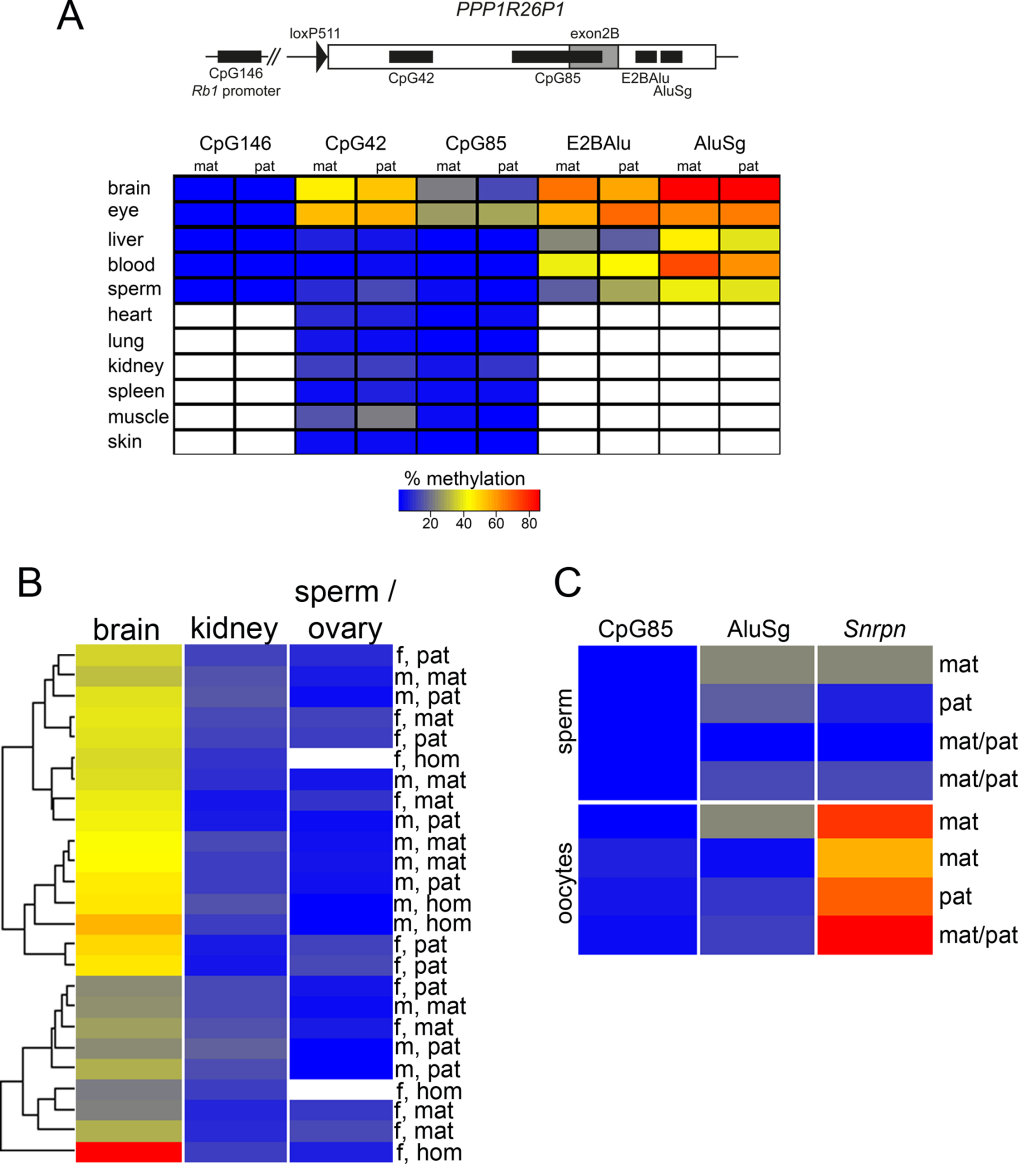
DNA methylation at PPP1R26P1 does not depend on parental inheritance. **A**) Top: Scheme of analyzed amplicons: the *Rb1* promoter CpG146, and CpG42, CpG85, E2BAlu, AluSg in *PPP1R26P1*. Bottom: Heat map of methylation levels at the five amplicons in all tissues analyzed. Results for two male mice are shown, one after maternal (mat) and one after paternal (pat) transmission of *PPP1R26P1*. White indicates not determined. Color key is for all figures. **B**) Heat map of methylation at CpG85 in 10 male (m) and 10 female (f) heterozygous mice, five after maternal (mat) and five after paternal (pat) transmission of the pseudogene. Results for mice homozygous (hom) for the pseudogene are included (three females, two males). The dendrogram shows the result of an unsupervised cluster analysis. **C**) Heat map of methylation in sperm and oocyctes; only samples with *Snrpn* methylation below 30% in sperm and above 50% in oocytes are shown. Full results of DNA methylation analyses in percent in all animals and all tissues are listed in S4 Table.

We analyzed DNA methylation of the CpG island at the *Rb1* promoter, CpG146, and four CpG-rich elements present in the integrated human *PPP1R26P1* pseudogene: CpG42, CpG85, AluSg and E2BAlu (Fig 2A top, chromosomal positions are listed in S2 Table) in heterozygous female and male mice, which inherited the human *PPP1R26P1* pseudogene either from the mother or the father. In humans, CpG42 is fully methylated on both parental alleles, whereas CpG85 is the DMR of human *RB1* and methylated on the maternal but not the paternal allele [16]. AluSg is an Alu element that integrated into *PPP1R26P1* after the Platyrrhini–Catarrhini split, since it is present in the human, chimpanzee, orang-utan and rhesus genome, but not in the marmoset [14]. Alu elements are repeat elements present in high copy number in the human genome and are inactivated by DNA methylation. They have been discussed to serve as seed for acquisition of DNA methylation [22, 23]. E2BAlu is a GC-rich region between the newly formed *RB1* exon 2B and the AluSg element. We analyzed this region to see how far DNA methylation would spread from AluSg towards CpG85.

The DNA methylation level of the described CpG-rich elements was analyzed in several tissues of heterozygous *Rb1_PPP1R26P1* mice by next-generation bisulfite sequencing using the Roche 454 GS Junior system and the Amplikyzer software for analysis [24]. To test for differences in DNA methylation due to parental transmission, we first analyzed tissues of two male mice, one after maternal and one after paternal transmission of *PPP1R26P1*, as shown in Fig 2A. Levels of DNA methylation in percent of all animals and tissues analyzed are listed in S4 Table. CpG146 in the *Rb1* promoter was unmethylated in brain, eye, liver, blood and sperm of both animals analyzed. DNA methylation at CpG42 was analyzed in all 11 tissues; brain, eye, heart, lung, kidney, spleen, liver, muscle, skin, blood, ovary and sperm cells. CpG42 showed variable methylation levels depending on the analyzed tissue. Highest levels (ranging from 43 to 56 percent) were observed in the tissues of neuroectodermal origin, brain and eye. All other tissues showed moderate to low levels of DNA methylation. The observed methylation levels were independent of the mode of transmission of the *PPP1R26P1* allele. DNA methylation of CpG85 was analyzed in all 11 tissues isolated. In the two male mice analyzed first, no difference in the level of CpG85 methylation was observed in respect to the mode of parental transmission. But as for CpG42, levels of DNA methylation at CpG85 were highest in brain and eye (ranging from 13 to 29 percent), and low in all other tissues analyzed (0.6 to 9 percent). DNA methylation of AluSg and E2BAlu was analyzed in brain, eye, liver, blood and sperm. In all tissues, these two regions showed higher levels of DNA methylation than CpG42 and Cp85, ranging from 16 to 67 percent for E2BAlu and from 38 to 86 percent for AluSg. Again, DNA methylation levels of both elements were highest in brain and eye.

Since we were most interested in DNA methylation of the human DMR CpG85, we extended the analysis to a total of 10 female and 10 male heterozygous *Rb1_PPP1R26P1* mice, with five individuals of maternal and paternal transmission for each sex (Fig 2B, S3 Table). In addition, methylation of CpG85 was analyzed in three female and two male mice homozygous for the *PPP1R26P1* allele. For this extended analysis, we focused on brain and kidney, representing tissues with higher and lower levels of methylation, and ovary and sperm, representing germ cell-containing tissue and germ cells, in which establishment of differential DNA methylation should occur. In all animals, methylation levels in brain (13 to 28 percent) were higher than in kidney (3 to 10 percent) and sperm or ovary (0.8 to 3 and 3 to 8 percent, respectively). To detect patterns, an unsupervised cluster analysis was performed with the CpG85 DNA methylation data of all 25 mice analyzed (Fig 2B). The DNA methylation levels did not cluster according to the mode of parental transmission of *PPP1R26P1*, showing that they are not dependent on the parental origin. Also, the mice homozyogous for *PPP1R26P1* did not cluster separately.

Methylation at DMRs of imprinted genes is set in one of the parental germ lines. As CpG85 did not show parent-of-origin-dependent methylation in any of the analyzed tissues, we were interested if methylation is not established in the germ line or if it is set but lost during development. Therefore, we investigated DNA methylation in oocytes and sperm. Analyzed loci included CpG85, AluSg and the maternally methylated DMR of the imprinted *Snrpn* locus on chromosome 7C as control. For sperm, two animals each were analyzed after maternal, paternal or homozygous transmission of *PPP1R26P1*. For oocytes, three animals each were analyzed after paternal, maternal and homozygous transmission of *PPP1R26P1*. *Snrpn* is a stable gametic DMR in human and mouse and is expected to show full methylation in oocytes and no methylation in sperm. In our analysis, we observed methylation levels at *Snrpn* ranging from 0.4 to 93.9% in oocytes and from 0.9 to 61.4% in sperm (S2 Fig, S4 Table). Because the deviant values may be attributed to contaminating surrounding cells or to technical difficulties in equal representation of a limited amount of DNA after the destructive bisulfite treatment, in Fig 2C only results are shown with DNA methylation levels below 30% in sperm and above 50% in oocytes at the *Snrpn* control locus. However, CpG85 was unmethylated in all sperm and oocyte samples analyzed, indicating that CpG85 does not get methylated in the germ line of either sex (Fig 2C, S2 Fig). In humans, Alu elements show higher methylation levels in oocytes than in sperm, but this could not be observed in our analysis in mice [25].

### Expression of the alternative transcript 2B

**Fig 3:**
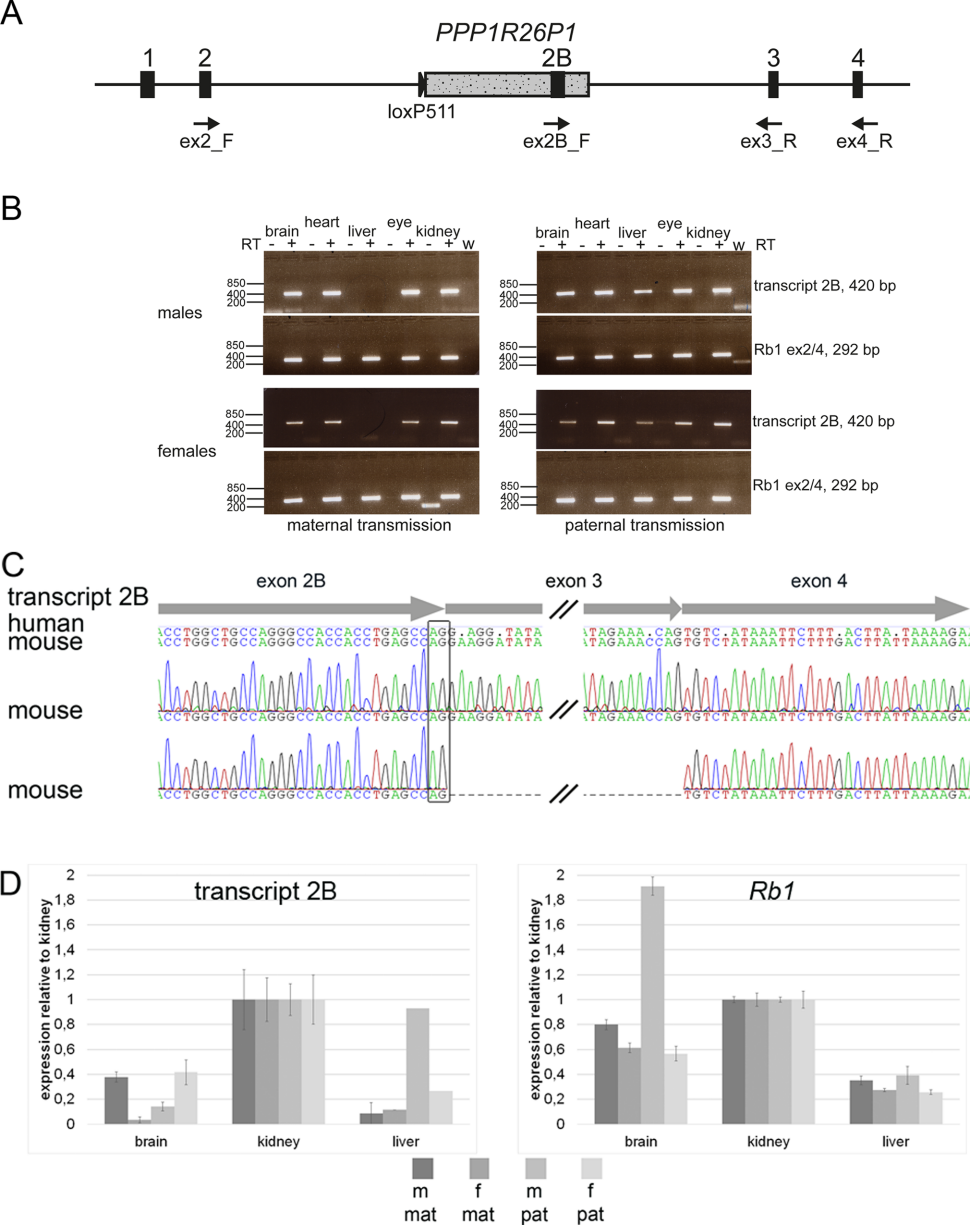
The alternative transcript 2B is expressed in *Rb1_PPP1R26P1* knock-in mice. **A**) Diagram of *Rb1_PPP1R26P1*. Positions, names and directions of RT-PCR primers are indicated. **B**) RT-PCR for transcript 2B (primers ex2B_F / ex3_R, size 420 bp) and *Rb1* (primers ex2_F / ex4_R, size 292 bp). +/- RT: with or without reverse transcriptase, w: water control. **C**) Sanger sequencing of RT-PCR products obtained by reverse priming in *Rb1* exon 4 and two rounds of amplification. The alternative exon 2B uses the same splice donor site than in humans, indicated by the black box. Exon structure of transcript 2B is shown with arrows at the top. Consensus sequences of human and mouse transcript 2B are shown with disagreements indicated by dots in the human sequence. Below, two electropherograms are shown of transcripts amplified in *Rb1_PPP1R26P1* knock-in mice, the lower showing exon skipping of *Rb1* exon 3. **D**) Quantitative RT-PCR of transcript 2B and *Rb1*. Mean and standard deviation of four technical replicates are shown. In liver, m/pat and f/pat showed a result for one of the replicates only. m, f: male or female; mat, pat: maternal or paternal transmission of *PPP1R26P1*.

In humans, expression of an alternative *RB1* transcript is observed from the unmethylated paternal allele, connecting the alternative exon 2B with exon 3 of *RB1* [16]. The definite length of the alternative transcript is not known yet. In murine ES cells, we observed transcriptional activity at CpG85, but we could not show the expression of a transcript connecting the alternative exon 2B with exon 3 of *Rb1* [20]. In heterozygous *Rb1_PPP1R26P1* mice we expect to observe expression of the normal *Rb1* transcript from both, the wildtype and the knock-in, alleles. In addition, if CpG85 acts as promoter in *Rb1*_*PPP1R26P1* mice, we expect to detect expression of an alternative *Rb1* transcript, starting at exon 2B in *PPP1R26P1* and splicing on to downstream exons, especially exons 3 and 4 of the *Rb1* gene. We examined expression of an alternative transcript 2B in brain, eye, heart, liver and kidney, representing tissues with different levels of DNA methylation. Expression was analyzed in four mice, two males and two females, one each after maternal or paternal transmission of the pseudogene. By RT-PCR, a transcript connecting exon 2B to mouse *Rb1* exon 3 was readily and constantly detected in all four mice in brain, heart, kidney and eye (Fig 3A and 3B). In liver, a transcript connecting exon 2B with exon 3 was detected only in animals that inherited the *PPP1R26P1* allele from the father (Fig 3A). Elongation of the alternative transcript at least up to exon 4 was detected in all tissues tested, brain, heart, liver and kidney, but only after two rounds of amplification (Fig 3A). Sequencing of the RT-PCR products revealed the use of the same splice donor site of exon 2B in mice as in humans (Fig 3B). Also, transcripts skipping exon 3 and directly connecting exon 2B to exon 4 were detected, indicating correct splicing from the new exon 2B to exon 4 of *Rb1* (Fig 3B).

We applied qPCR to determine whether transcript levels correlate with DNA methylation, expecting lower transcript levels in tissues with higher methylation of CpG85 (Fig 3C). Brain was used, representing the tissue with the highest level of DNA methylation observed at CpG85, as well as kidney and liver, representing tissues with low methylation at CpG85. Again, four heterozygous *Rb1_PPP1R26P1* mice were included in the analysis, two males and two females, one each after maternal or paternal transmission of the pseudogene. Expression levels observed for the alternative *Rb1* transcript 2B were about twice as high in kidney as in brain. In liver, expression levels of transcript 2B were lower than in kidney, although the level of DNA methylation at CpG85 was equally low in both tissues. The expression level of the full length *Rb1* transcript was also lower in liver than in brain or kidney.

### Skewing of *Rb1* expression

**Fig 4:**
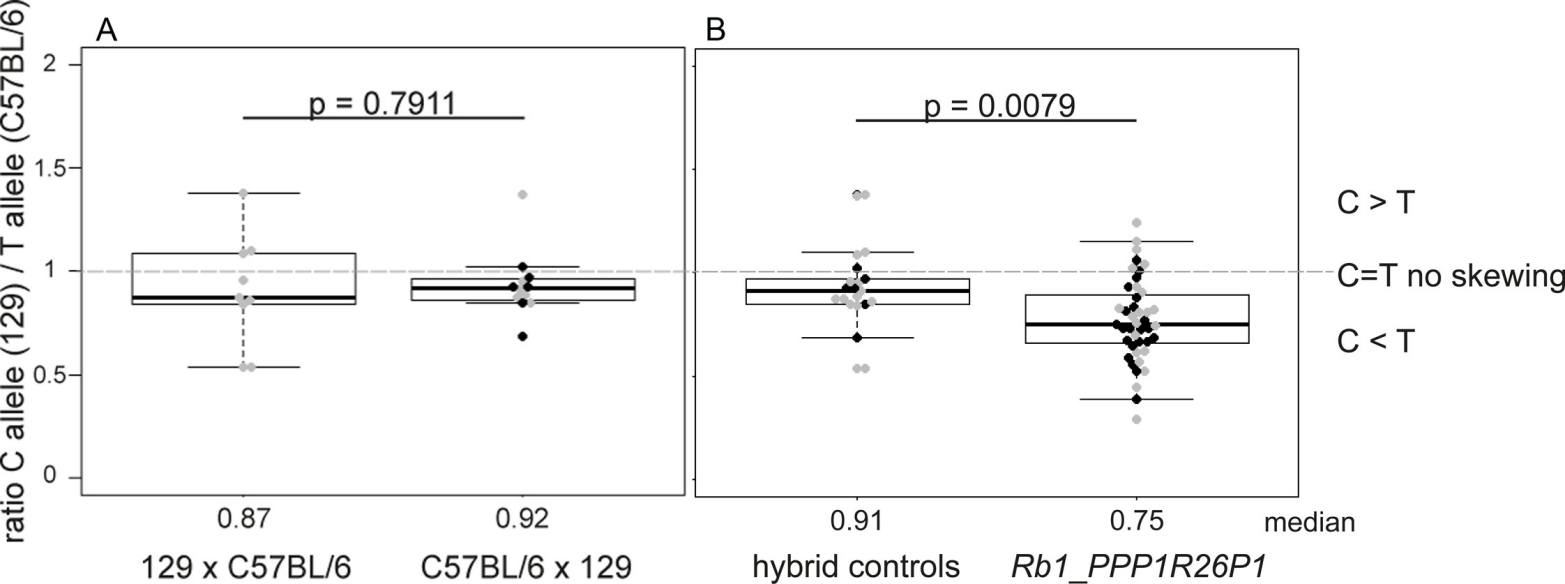
*Rb1* expression is repressed in cis in *Rb1_PPP1R26P1* heterozygous mice. **A**) Hybrid control breeding of C57BL/6J and 129 animals in both directions. The maternal genotype is always written on the left side. Expression was analyzed in brain, kidney and liver. Results of all tissues are pooled in one boxplot. **B**) Comparison of allelic *Rb1* expression in hybrid control breeding (pooled results from A) with *Rb1*_*PPP1R26P1* mice. Analysis was performed after maternal or paternal transmission of *PPP1R26P1* in brain, kidney and liver; all measurements are depicted in one boxplot. *Rb1*_*PPP1R26P1* mice show significant skewing in favor of the wildtype C57BL/6J (T) allele. Grey dots: female animlas, black dots: male animals. Bee swarm boxplots show the median (given in numbers below the diagram), the quartiles and p-values were calculated using Welch’s t-test.

In humans, *RB1* expression is higher from the maternal allele, where the CpG85 promoter is methylated and silenced, than from the paternal allele, where the CpG85 promoter is active and expresses the alternative transcript 2B [16]. In murine ES cells, we observed higher expression from the non-modified, wildtype allele compared to the allele carrying the *PPP1R26P1* insertion in an unmethlyated state [20]. Thus, presence of unmethylated CpG85 negatively influences *RB1/Rb1* expression in cis. Having observed unmethylated CpG85 and expression of the alternative *Rb1* transcript in different tissues of *Rb1_PPP1R26P1* mice, we examined if it influences *Rb1* transcription. To do so, expression of both *Rb1* alleles was analyzed by single nucleotide primer extension using the single nucleotide polymorphism rs13465574 (T/C) in exon 25 of the *Rb1* gene, which is present in mouse strains C57BL/6J (T allele) and 129 (C allele) (Fig 4). *Rb1_PPP1R26P1* mice carry a wildtype *Rb1* T allele of the C57BL/6J strain used for backcrossing, and the *Rb1* knock-in C allele inherited from the modified ES cells of mouse strain 129 used for blastocyst injection. To test for strain-specific differences in strength of *Rb1* expression, breeding of wildtype C57BL/6J and 129 animals was set up. Three animals, one female and two males, with a C57BL/6J mother and a 129 father were analyzed (C57BL/6 x 129; note the maternal allele is always on the left). In addition, two female animals from a 129 mother and a C57BL/6J father (129 x C57BL/6) were analyzed. Allelic *Rb1* expression was analyzed in brain, kidney and liver. Presence of alleles C and T were measured in genomic DNA and cDNA (representing mRNA transcript levels) and the C/T ratios were calculated. The RNA ratio was normalized to the DNA ratio, assuming that both alleles have a ratio of one on DNA level. Therefore, a value of about one indicates no skewing in expression of the 129 (C) and the C57BL/6J (T) allele. A value above one indicates skewing in favor of the 129 (C) allele and a value below one skewing in favor of the C57BL/6J (T) allele. Since we did not assume a difference in allelic *Rb1* expression in different tissues, resulting ratios of all samples were pooled (Fig 4). In the hybrid breeding, no expression bias was observed towards one of the two alleles, indicating that in the wildtype, expression of the C allele is as strong as the expression of the T allele (Fig 4A). In heterozygous *Rb1*_*PPP1R26P1* mice, allelic expression was analyzed in the tissues brain, kidney and liver as well. Four females and four males each were analyzed for paternal and maternal *PPP1R26P1* transmission, resulting in eight animals per group. Detailed analyses of modes of transmission in single tissues yielded significant results for liver after maternal inheritance of *PPP1R26P1* and for brain after maternal and paternal inheritance (S3B Fig). All other results were not significant, indicating that there are only little differences in allelic expression levels of *Rb1* between different tissues and no dependency on parental transmission of the *PPP1R26P1* allele. However, a pooled analysis of ratios obtained in all animals and tissues analyzed shows highly significant skewing of *Rb1* expression in favor of the non-modified C57BL/6J T allele compared to the pooled wildtype hybrid animals (Fig 4B). This finding indicates a cis-repressive influence of *PPP1R26P1* on *Rb1* expression in mice, as has already been described in mESCs [20].

## Discussion

To study the induction of genomic imprinting of mouse *Rb1* by introduction of a proven human gDMR, we generated *Rb1_PPP1R26P1* knock-in mice to pass the human *PPP1R26P1* pseudogene through the mouse germ line. Our results show that human CpG85 does not function as gDMR in the mouse, but acquires tissue-specific DNA methylation in cells of the neuroectodermal lineage. Promoter function of the unmethylated CpG85 and the function of *PPP1R26P1* as cis-repressive element for full-length *Rb1* transcription are maintained in the mouse.

### Tissue-specific methylation of CpG85

Analysis of DNA methylation was carried out in tissues of heterozygous animals with only one copy of *PPP1R26P1* per cell. This means with an observed total methylation of 30%, a methylated allele was present in 30% of cells, whereas the allele was not methylated in 70% of cells. We observed tissue-specific methylation of CpG85 and CpG42 in brain and eye. Both tissues are of neuroectodermal origin. The levels of methylation at CpG42 ranged from 43 to 52 percent in brain and from 53 to 56 percent in eye. For CpG85, we observed methylation levels from 13 to 28 percent in brain and from 25 to 29 percent in eye. For both CpG islands, the methylation level of all analyzed animals was consistently higher in eye compared to brain. In brain, DNA methylation levels varied considerably between animals. Since we used random parts of the brain and the complete eye for DNA preparation, the samples represented a pool of different cell types, which might have disparate methylation levels. The use of a random pool of cells for analysis could explain the variation of DNA methylation levels between animals.

Tissue-specific DNA methylation has a function during cell differentiation and is thought to influence gene expression. Usually, DNA methylation correlates with gene suppression [26]. We and others showed that promoter activity at CpG85 leads to suppression of *Rb1* transcription in cis [16, 20, 27]. Thus, cells with a *PPP1R26P1* allele that are unmethylated at CpG85 are expected to have a higher level of transcript 2B expression resulting in a lower expression of full-length *Rb1*. Methylated CpG85 should correlate with low expression of transcript 2B and higher expression of full-length *Rb1*. We used kidney and brain tissue for analysis of quantitative expression, because kidney is unmethylated at CpG85 and brain shows methylation levels between 13 to 28 percent. The expression level of transcript 2B in brain was at least 2.5-fold lower than in kidney (Fig 3C), indicating that expression of transcript 2B correlates with DNA methylation. However, full-length *Rb1* expression is also lower in brain than in kidney. Comparing liver to kidney, we would have expected only slight differences, since both tissues show a low level of DNA methylation at CpG85. But in liver, levels of transcript 2B and *Rb1* were significantly lower than in kidney and even lower than in brain (Fig 3C). Kanber et al. could not detect a transcript 2B in human liver, pointing to low levels of expression in this tissue [16]. Interestingly, a report about hepatocellular carcinoma easily detected transcript 2B in liver [27]. These and our data indicate that methylation of CpG85 might not be the only regulator of *Rb1* and transcript 2B expression.

We observed highest levels of DNA methylation at *PPP1R26P1* in brain and eye, representing tissues of neuroectodermal origin (Fig 2A). Also, we showed that insertion of (unmethylated) *PPP1R26P1* leads to suppression of *Rb1* expression in cis (Fig 4). This suggests a need for a higher level of *Rb1* expression in neuroectodermal tissue. The essential role for *Rb1* in the retina, a tissue of neuroectodermal origin, is documented by the development of retinoblastoma upon biallelic inactivation of *RB1* in humans [28]. In mice, inactivation of *Rb1* is necessary but not sufficient for the development of retinoblastoma [29–32].

### *PPP1R26P1* does not acquire differential methylation in the mouse

Many imprinted genes and gene clusters are conserved between mouse and human in organization and regulation, indicating that the machinery for imprint erasure, establishment and maintenance is conserved between the two species [1]. Our methylation analysis of CpG85 in oocytes and sperm shows that this CpG island is completely unmethylated in both types of germ cells, indicating that the sequence is not recognized by the imprint establishment machinery in the mouse. Imprint setting was thought to work by targeting methylation only to specific sequences, the gDMRs. Recent work suggests that imprinted gDMRs are not treated differently than other sequence elements during establishment of DNA methylation, but that the difference lies in maintenance during the genome reprogramming phase after fertilization (reviewed in [33, 34]). For establishment of DNA methylation at gDMRs, transcription and recruitment of de novo DNA methyltransferases (DNMTs) are needed. DNMTs are recruited by specific histone marks, and the type of histone marks are connected to transcriptional activity of a gene. An active gene is marked by tri-methylation of lysine 4 of histone 3 (H3K4me3) at the promoter and by tri-methylation of lysine 36 of histone H3 (H3K36me3) in the gene body [35, 36]. DNMT3L, the accessory factor of DNMT3A, is repelled by H3K4me3, making sure that active promoters stay unmethylated [37, 38]. In contrast, DNMT3A is directly recruited to gene bodies by H3K36me3, leading to DNA methylation at CpG sites in active transcription units [39, 40]. Transcriptional read-through over gDMRs has been observed in oocytes and sperm and has been correlated with acquisition of DNA methylation [6, 41, 42]. In oocytes, the maternally methylated gDMRs of the *Snrpn* locus (PWS-SRO) and the *Gnas* locus have been shown to be covered by transcriptional read-through initiating at upstream, oocyte-specific promoters, and this transcription proved to be necessary for establishment of DNA methylation [43–45]. Transcriptional read-through has also been observed at the paternally methylated gDMRs at the *H19* gene and IG-DMR in sperm but not in oocytes [41]. Genomic organization of CpG85 is similar in humans and in the *Rb1_PPP1R26P1* mice: CpG85 is located in intron 2 of the *RB1*/*Rb1* gene. Because CpG85 is a maternally methylated DMR in humans, we assume that it is part of an active transcription unit in human oocytes. Publicly available datasets of high-throughput RNA sequencing in human oocytes indicate that the *RB1* gene is transcribed at low levels [46, 47]. Absence of transcription of the *Rb1* gene in mouse oocytes could provide and explanation for lack of DNA methylation on CpG85. However, datasets of RNA sequencing in mouse oocytes also indicate expression of *Rb1*, excluding missing transcription for failure of DNA methylation establishment on CpG85 in mouse oocytes [46, 48].

### Influence of the surrounding sequence

DNA methylation has been described as a mechanism of host defense, silencing transposable DNA elements to prevent harm to the genome [49, 50]. This mechanism has been used to explain the observed silencing of randomly integrated transgenes [51]. Usually, transgenes carry a reasonably strong promoter and integrate in multiple copies, and it is unclear whether this contributes to recognition of the transgene by genome surveillance mechanisms, resulting in their silencing. The introduced CpG85 did not acquire methylation in mouse germ cells and most differentiated tissues (Fig 2A), indicating that single copy human *PPP1R26P1*, carrying a weak promoter, is not recognized as foreign DNA that has to be inactivated by DNA methylation in the mouse genome.

In multiple transgene studies, imprinted silencing of the transgene has been observed in one of several generated transgenic lines, suggesting that imprinted transgene silencing depends on the position of genomic integration. [52, 53]. In all but one case, imprinted transgenes were exclusively or preferentially methylated upon maternal but not paternal transmission. However, these studies did not investigate if transcriptional read-through of imprinted transgenes occurs at the integration site. The dependency of imprinted transgene silencing on the site of integration indicates an influence of the surrounding sequence on the transgene. This could also play a role in evolution and acquisition of differential methylation at CpG85 in *PPP1R26P1*. The analysis of four other retrocopies of *PPP1R26*, which integrated at different positions on human chromosome 22, revealed another fate than that of the retrocopy that integrated into the *RB1* gene [14, 16]. The four copies on chromosome 22 are integrated in intergenic regions instead of within a host gene, and CpG islands corresponding to CpG42 and CpG85 in *PPP1R26P1* did not evolve from the CpG-rich regions of these copies. So far, investigations have also shown that the *RB1* locus is unique in becoming imprinted by intragenic integration of a pseudogene. Of 104 intronic CpG islands, which have been identified in humans but not in mice and which have a suggestive origin in a retrotransposition event, none showed parent-of-origin-dependent acquisition of DNA methylation [54].

## Conclusion

In conclusion, our results of the humanized mouse model of *RB1* imprinting demonstrate that introducing a proven human gDMR into the respective genomic position in the mouse does not necessarily result in imprinting of the respective mouse gene. This is true, even though CpG85 exhibits promoter function and repression of the main *Rb1* transcript in the mouse, as it was described in the human. Our results indicate that the *PPP1R26P1* sequence is not recognized by the imprint establishment mechanisms in the mouse germ line and that this might be due to the pivotal role of the surrounding sequence in induction of DNA methylation. Conservation of intronic sequences between species is generally lower than that of exons. In intron 2 of the human and mouse *RB1/Rb1* gene, only two small evolutionary conserved regions have been identified and the overall conservation of human and mouse intron 2 of the *RB1/Rb1* genes is low [20]. It would be of interest to determine the elements present in human *RB1* intron 2 but absent in mouse *Rb1* intron 2, which are necessary and sufficient to induce differential methylation of CpG85 in the female germ line. Despite the failure of imprint establishment in the mouse germ line, our mouse model opens the possibility to investigate the cellular function of the alternative *Rb1* transcript 2B, which is expressed from the unmethylated CpG85 promoter. This includes the determination of the final length of the alternative transcript and the possible expression of a truncated *Rb1* protein. These points, which could be relevant for the development of retinoblastoma tumors, are not yet addressed in humans, because of lack of a suitable experimental system.

## Methods

### Modification of murine ES cells

All media and supplements for cell culture were from Life Technologies, unless otherwise stated. Male R1 mouse ES cells of strain 129 (received May 2012, provided by R. Waldschütz, Central Animal Facility, University Hospital Essen) were cultivated on mouse inactivated feeder cells in Knockout DMEM (#10829018) supplemented with 20% fetal calf serum (PAA, #A15-151), 1% glutamine (#25030024), 1% non-essential amino acids (#11140035), 1% penicillin/streptomycin (#15140122), 2 μM β-mercaptoethanol (#31350010) and 1/1000 LIF in a humidified incubator at 37°C and 5% CO_2_. Sub-culturing was performed every second to third day. Gene targeting was performed by electroporating 5x10^6^ cells with 50 μg of linearized targeting vector using a BioRad GenePulser at 500 μF and 0.24 kV. The targeting vector contained a 6.6 kb homology fragment of mouse *Rb1* intron 2 (Fig 1A; chromosomal positions in S2 Table), into which the human pseudogene sequence *PPP1R26P1* of 5.2 kb and a neomycin selection cassette were inserted (for details see [20]). Cells were plated on a resistant feeder layer and selection was performed for seven days using 200 μg/ml G418 (#10131035). Single clones were picked, expanded and screened for *PPP1R26P1* integration by Southern blot.

### Karyotyping of murine ES cells

For karyotyping, cells were seeded on gelatine and passaged three times before harvesting. After reaching 50 to 60% confluence, cells were treated with 0.1 μg/ml colcemid (Roche, #10295892001) for 2 hours in the incubator. Cells were trypsinized and transferred to a 15 ml Falcon tube and collected by centrifugation at 200 x *g* for 4 min. Hypotonic treatment in 0.4% KCl was performed for 10 min at 37°C in a water bath. At the end of hypotonic treatment, 100 μl of fresh-made fixative (3:1 methanol:acetic acid) was added and mixed by inversion. Cells were collected by centrifugation at 200 x *g* for 4 min and 5 ml of fixative was added to the pellet for 20 min at room temperature. After centrifugation the pellet was resuspended in 500μl of fresh-made fixative. Two drops were spotted on a pre-chilled slide for a quality check under the microscope. At least 15 metaphases each were counted for the seven independent clones. Four clones had the correct number of 40 chromosomes (S1 Table).

### Southern blot

Southern blotting was performed as described in Steenpass et al. [20]. Briefly, genomic DNA was digested with BamHI (NEB, #R3136L) separated by gel electrophoresis and blotted by capillary transfer using standard procedures [55]. The KIAA 5’ext probe was randomly labelled with ^32^P-α-dCTP using the Megaprime kit (GE, #RPN1607) and purified using the QIAquick Nucleotide Removal Kit (Qiagen, #28304). Overnight hybridization and washing of blots was performed at 65°C in Church buffer (7% SDS, 1 mM EDTA, 0.5 M NaPO_4_, pH 7.4).

### Generation and breeding of mice

This study was carried out in strict accordance with the recommendations in the Guide for the Care and Use of Laboratory Animals of the German Government. This study and the generation of knock-in mice were approved by the Committee on the Ethics of Animal Experiments (*Landesamt für Natur*, *Umwelt und Verbraucherschutz*, LANUV AZ 84–02.04.2012.A168). Mice were kept under SPF conditions (12 hours light and dark cycle, food and water ad libitum, standard blue line cage) in the Central Animal Facility of the University Hospital Essen.

Cells of mESC R1 clones 24 and 42 were thawed and injected into 53 and 66 blastocysts, respectively, of C57BL/6J origin. Six to 20 blastocysts were transferred to one C57BL/6J foster mouse. In total, 10 foster mice were transplanted. Seven chimeras were born carrying the genetic material of clone 24 and 17 chimeras that of clone 42. Chimerism was determined visually by coat color. Chimerism ranged from 10 to 50% for clone 24 and between 30 and 90% for clone 42.

For each line, the three best chimeric males with highest grade of chimerism were crossed with C57BL/6J females to test for germline contribution of the injected ES cells. For clone 24, no offspring with agouti coat color was obtained, indicating failure of germ line contribution of injected ES cells. For clone 42, three agouti animals were obtained and backcrossed to a C57BL/6J background. The generation N1 was crossed with mice of the CMV-Cre deleter strain, which carry the transgene Tg(CMV-Cre)1Cgn on the X chromosome (females are heterozygous) [21]. This transgene is expressed in all types of cells including germ cells and the zygote and leads to removal of the neomycin selection cassette in the zygote. Tail snips were taken for genotyping in generations F0 to N4, ear snips were used from N5.

Microsatellite screening was performed to test for the degree of backcrossing of the modified *PPP1R26P1* allele into a C57BL/6J background. Multiplex PCR was performed using two markers per autosome and one marker for the X chromosome, loci and primer sequences are available upon request [56]. According to this analysis, backcrossing was complete in generation N5.

### Study design

Genomic imprinting relates to parental inheritance of the *Rb1_PPP1R26P1* allele. The study contained three groups: 1) animals having inherited the *Rb1_PPP1R26P1*allele from the mother (Rb1_PPP1R26P1 / +), 2) animals having inherited the *Rb1_PPP1R26P1* allele from the father (+/*Rb1_PPP1R26P1* ***)***, 3) animals being homozygous for *Rb1_PPP1R26P1* having inherited one allele from each parent. All groups contained male and female animals in same numbers, aged between eight weeks and three months.

Animals were sacrificed and organs prepared for isolation of genomic DNA and RNA.

### Preparation of mouse organs

Mice were sacrificed by cervical dislocation after anesthesia using isoflurane. Organs were prepared in the following order: sperm, spleen, kidney, liver, lung, heart, blood, skin, muscle, brain, eyes. Organs were cut into three pieces, snap frozen in liquid nitrogen and stored at -80°C until RNA or DNA preparation.

Sperm was isolated by preparation of the *vas deferens* and *cauda epididymis*, which were placed into pre-warmed PBS. Sperm in the *vas deferens* was streaked out using a spatula and the *cauda epididymis* was cut to allow sperm to swim up during the following incubation at 37°C for 20 min. Afterwards, PBS with sperm was collected, sperm was pelleted by centrifugation and the supernatant removed. The pellet was snap frozen in liquid nitrogen and stored at -80°C until preparation.

Oocytes were obtained by superovulation after hormonal stimulation of female mice. Oocyte maturation was induced by i.p. injection of 10 IU pregnant mare’s serum gonadotropin (PMSG, Intervet). Ovulation was induced 48 hours later by i.p. injection of 10 IU human chorionic gonadotropin (hCG, Intervet). After 15–17 hours, cumulus-oocyte-complexes were isolated from the oviductal ampullae. Cumulus cells were removed by digestion with hyaluronidase and subsequent washings in M2 medium (Sigma, #M7167). Metaphase II oocytes were immediately frozen at -80°C until further use.

### Preparation of genomic DNA and RNA

Standard procedures were used to isolate genomic DNA by alkaline lysis followed by precipitation [55]. This method was used to prepare DNA from confluent R1 ES cell clones, mouse tail snips and mouse organs. Tail snips and organs were digested overnight with proteinase K at 56°C [55]. Mouse ear snips were digested in a PCR lysis buffer (1x PCR buffer without MgCl_2_, 0.5 mM MgCl_2_, 0.045% Tween 20, 0.045% NP-40, 50 μg/ml Proteinase K) for at least 2 hours and then directly subjected to genotyping PCR.

Total RNA from mouse organs was prepared using Qiazol (Qiagen, #79306) according to manufacturer’s instructions, after homogenization in a FastPrep machine using Lysing matrix D beads (MP Biomedicals, #6913–100).

### Next-generation bisulfite amplicon sequencing

For bisulfite conversion of 9 to 40 oocytes, the EZ DNA Methylation-Direct kit (Zymo Research, #D5020) was used according to protocol. After bisulfite conversion, an outer PCR was performed, using GoTaq HotStart Green MasterMix (Promega, #M7422) and 2 μl of bisulfite-converted DNA. General PCR conditions were: 95°C for 2 min, (95°C for 30 sec, annealing temperature as in S5 Table for 30 sec, 72°C for 45 sec) 35 cycles, 72°C for 7 min, 4°C hold. Of this PCR product, 3 μl were used for a nested inner PCR reaction with GoTaq HotStart Green MasterMix and tagged primers specific for the amplicons studied.

For all other organs, 500 ng of genomic DNA was converted using the EZ DNA Methylation-Gold kit (Zymo Research, #D5006), according to the protocol. Bisulfite-converted DNA (1 μl) was used for a single PCR reaction using the GoTaq HotStart Green MasterMix and tagged, amplicon-specific primers as described for the nested oocyte PCR.

In a next PCR, multiplex identifier (MID) barcodes were added to the amplicons of oocytes and all other organs, using primers binding to the tag sequences added in the previous PCR. Samples were purified, enriched, pooled and processed for sequencing on the Roche 454 GS Junior platform as described previously [57]. Data analysis after the run included quality filtering and processing using the Amplikyzer software [24]. In brief, the Amplikyzer software sorts reads according to MIDs and amplicon sequences and performs methylation analysis of CpG sites present in the amplicons. The bisulfite conversion rate has to be at least 95% for reads to be considered. Results are depicted as methylation heat maps showing CpG sites in columns with one read per row. Methylation per CpG position and mean overall methylation percentage is given. All PCR primers and annealing temperatures are listed in S5 Table. MID primers are constructed containing a 21 bp sequencing adapter, a 4 bp key, a 10 bp mulitplex identifier (MID) and a 18 bp tag complementary to tags of the amplicons. Tag-sequences are: forward tag 5’-cttgcttcctggcacgag-3’ and reverse tag 5’- caggaaacagctatgac-3’. One example of an MID primer is given in S5 Table. A full MID primer list is available on request.

### RT-PCR and qRT-PCR

For transcript analysis, 1 μg of RNA was treated with RQ1 RNase-free DNaseI (Promega, #M6101) and reverse transcribed into cDNA using the components of the GeneAmp RNA PCR core kit (Life Technologies, #N8080143) in a total volume of 20 μl. For detection of transcript 2B, 10 μl cDNA was used per PCR reaction using GoTaq HotStart Green MasterMix (Promega, #M7422). Only 1 μl was used for the control reaction. The PCR protocol was as follows: 95°C for 2 min, (95°C for 30 sec, annealing temperature according to S5 Table for 30 sec, 72°C for 30 sec) 50 cycles, 72°C for 7 min, 4°C hold.

Quantitative real-time PCR (qRT-PCR) was performed using LightCycler480 Probes MasterMix (Roche, #04707494001) and primers with respective UPL probes of the Roche universal probe library. The same sample input was used for qRT-PCR as described above for RT-PCR. All samples were analyzed in four technical replicates on the Roche LightCycler 480II, using following amplification protocol: 95°C for 10 min, (95°C for 10 sec, 60°C for 30 sec, 72°C for 1 sec) 50 cycles, 40°C for 30 sec. Primer sequences and UPL probe numbers are listed in S5 Table.

### SNaPshot analysis

The ABI Prism SNaPshot ddNTP Primer extension kit (Life Technologies, #4323159) was used for determination of allelic ratios of mRNA transcripts (after reverse transcription into cDNA) as stated in the manufacturer’s instructions. Genomic DNA of the same sample tissue was used as reference. The reaction products were analyzed on an ABI 3130XL sequencer (Applied Biosystems) and electropherograms were analyzed using Gene Mapper 4.0 software (Applied Biosystems). Allelic ratios of cDNA were normalized to ratios obtained with genomic DNA from the same sample. Data are presented as bee swarm boxplots calculated using the respective packages provided by The R Project for Statistical Computing. In the boxplots, the median and quartiles are indicated. The p-values were calculated in R using Welch’s unequal variances t-test. Primer sequences are listed in S5 Table.

## Supporting Information

S1 Checklist ARRIVE checklist. (PDF)

S1 Fig Breeding scheme of the *Rb1*_*PPP1R26P1* knock-in mice. Injection of ES cells into blastocysts resulted in F0 chimeras. Male chimeras were mated with wildtype C57BL/6J females to start backcrossing on C57BL/6J and to test for germ line transmission in N1. N1 animals were mated with animals of the CMV-Cre deleter strain for removal of the neomycin selection cassette in the subsequent generation N2. Offspring of the next breeding of N2 animals to wildtype C57BL/6J animals was screened for loss of the Cre transgene and further bred to C57BL/6J background. Animals in generations N4 and N5 were used for the analyses. Backcrossing was complete in generation in N5.
(PDF)

S2 Fig CpG85 is unmethylated in sperm and oocytes. Heat map of methylation levels measured at CpG85, the AluSg element and *Snrpn*. CpG85 is completely unmethylated in sperm and oocyes and AluSg shows low to moderate levels of DNA methylation in both types of germ cells, ranging from 0 to 40%. Levels of methylation at *Snrpn*, expected to be free of methylation in sperm and fully methylated in oocytes, range from 0.4 to 93.6%. Mat: maternal transmission of *PPP1R26P1*, pat: paternal transmission, mat/pat: animals homozygous for *PPP1R26P1*.
(PDF)

S3 Fig Detailed results of SNaPshot analyses. A) Level of allelic *Rb1* expression in heterozygous *Rb1_PPP1R26P1* mice either after maternal (left) or paternal (right) transmission of the pseudogene, compared to the respective wildtype control. Results of all tissues are included in one plot. B) Level of allelic *Rb1* expression depicted per tissue and per type of transmission of the pseudogene. Grey: female animals, black: male animals. P-values were calculated using Welch’s t-test.
(PDF)

S1 Table Result of karyotyping of eight correctly targeted ES cell clones. (PDF)

S2 Table Chromosomal positions of analyzed regions. (PDF)

S3 Table Summary of DNA methylation analyses in *PPP1R26P1* heterozygous mice. CGI: CpG island analyzed, m, f: male or female; mat, pat: maternal or paternal inheritance of *PPP1R26P1*. Br: brain, li: liver, sp: spleen, ki: kidney, lu: lung, he: heart, sk: skin, mu: skeletal muscle.
(PDF)

S4 Table Results of DNA methylation analyses in percent. m, f: male or female; mat, pat: maternal or paternal transmission of *PPP1R26P1*; SPxx: identification numbers of mice.
(PDF)

S5 Table Primer sequences. (PDF)
