# Supplementary material for: A Mouse Model for Imprinting of the Human Retinoblastoma Gene
Source: PLoS One. 2015 Aug 14;10(8):e0134672. doi: 10.1371/journal.pone.0134672 (PMC4537222; doi:10.1371/journal.pone.0134672)
Supplement: S1 Fig — Injection of ES cells into blastocysts resulted in F0 chimeras. Male chimeras were mated with wildtype C57BL/6J females to start backcrossing on C57BL/6J and to test for germ line transmission in N1. N1 animals were mated with animals of the CMV-Cre deleter strain for removal of the neomycin selection cassette in the subsequent generation N2. Offspring of the next breeding of N2 animals to wildtype C57BL/6J animals was screened for loss of the Cre transgene and further bred to C57BL/6J background. Animals in generations N4 and N5 were used for the analyses. Backcrossing was complete in generation in N5. (PDF) [file pone.0134672.s002.pdf]

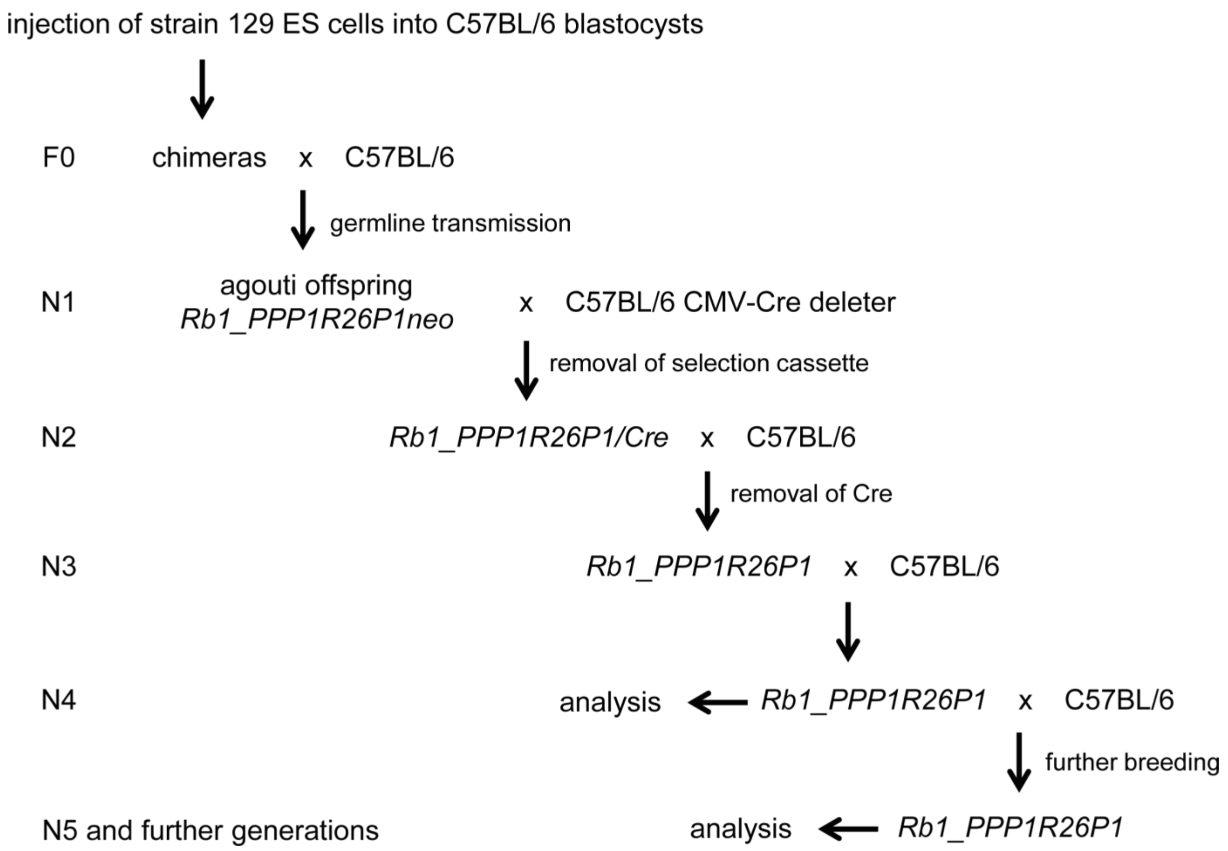

S1 Fig.: Breeding scheme of the *Rb1_PPP1R26P1* knock-in mice. Injection of ES cells into blastocysts resulted in F0 chimeras. Male chimeras were mated with wildtype C57BL/6J females to start backcrossing on C57BL/6J and to test for germ line transmission in N1. N1 animals were mated with animals of the CMV-Cre deleter strain for removal of the neomycin selection cassette in the subsequent generation N2. Offspring of the next breeding of N2 animals to wildtype C57BL/6J animals was screened for loss of the Cre transgene and further bred to C57BL/6J background. Animals in generations N4 and N5 were used for the analyses. Backcrossing was complete in generation in N5.
