# Supplementary material for: A Mouse Model for Imprinting of the Human Retinoblastoma Gene
Source: PLoS One. 2015 Aug 14;10(8):e0134672. doi: 10.1371/journal.pone.0134672 (PMC4537222; doi:10.1371/journal.pone.0134672)
Supplement: S2 Fig — Heat map of methylation levels measured at CpG85, the AluSg element and Snrpn. CpG85 is completely unmethylated in sperm and oocyes and AluSg shows low to moderate levels of DNA methylation in both types of germ cells, ranging from 0 to 40%. Levels of methylation at Snrpn, expected to be free of methylation in sperm and fully methylated in oocytes, range from 0.4 to 93.6%. Mat: maternal transmission of PPP1R26P1, pat: paternal transmission, mat/pat: animals homozygous for PPP1R26P1. (PDF) [file pone.0134672.s003.pdf]

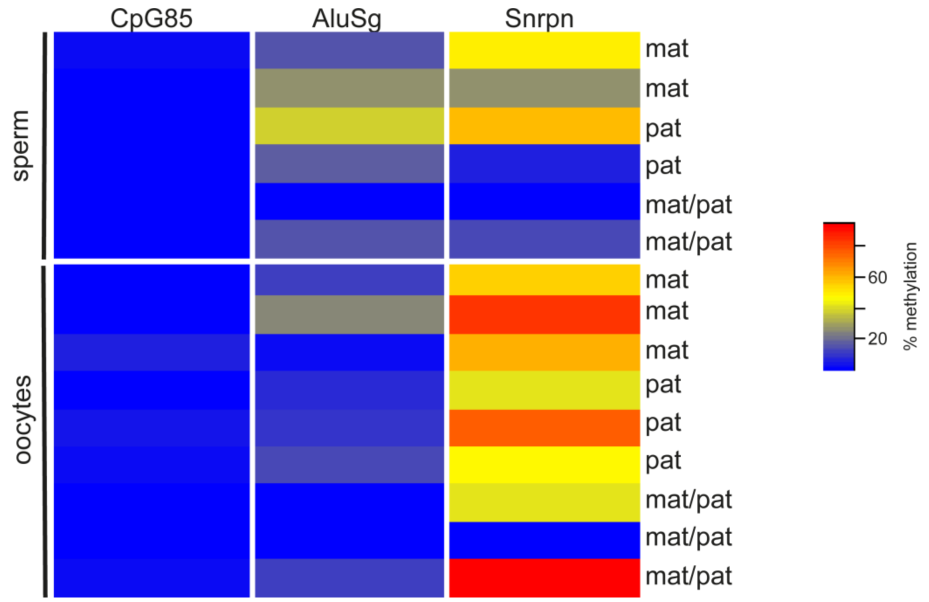

S2 Fig.: CpG85 is unmethylated in sperm and oocytes. Heat map of methylation levels measured at CpG85, the AluSg element and *Snrpn*. CpG85 is completely unmethylated in sperm and oocyes and AluSg shows low to moderate levels of DNA methylation in both types of germ cells, ranging from 0 to 40%. Levels of methylation at *Snrpn*, expected to be free of methylation in sperm and fully methylated in oocytes, range from 0.4 to 93.6%. Mat: maternal transmission of *PPP1R26P1*, pat: paternal transmission, mat/pat: animals homozygous for *PPP1R26P1*.
