# Supplementary material for: A Mouse Model for Imprinting of the Human Retinoblastoma Gene
Source: PLoS One. 2015 Aug 14;10(8):e0134672. doi: 10.1371/journal.pone.0134672 (PMC4537222; doi:10.1371/journal.pone.0134672)
Supplement: S3 Fig — A) Level of allelic Rb1 expression in heterozygous Rb1_PPP1R26P1 mice either after maternal (left) or paternal (right) transmission of the pseudogene, compared to the respective wildtype control. Results of all tissues are included in one plot. B) Level of allelic Rb1 expression depicted per tissue and per type of transmission of the pseudogene. Grey: female animals, black: male animals. P-values were calculated using Welch’s t-test. (PDF) [file pone.0134672.s004.pdf]

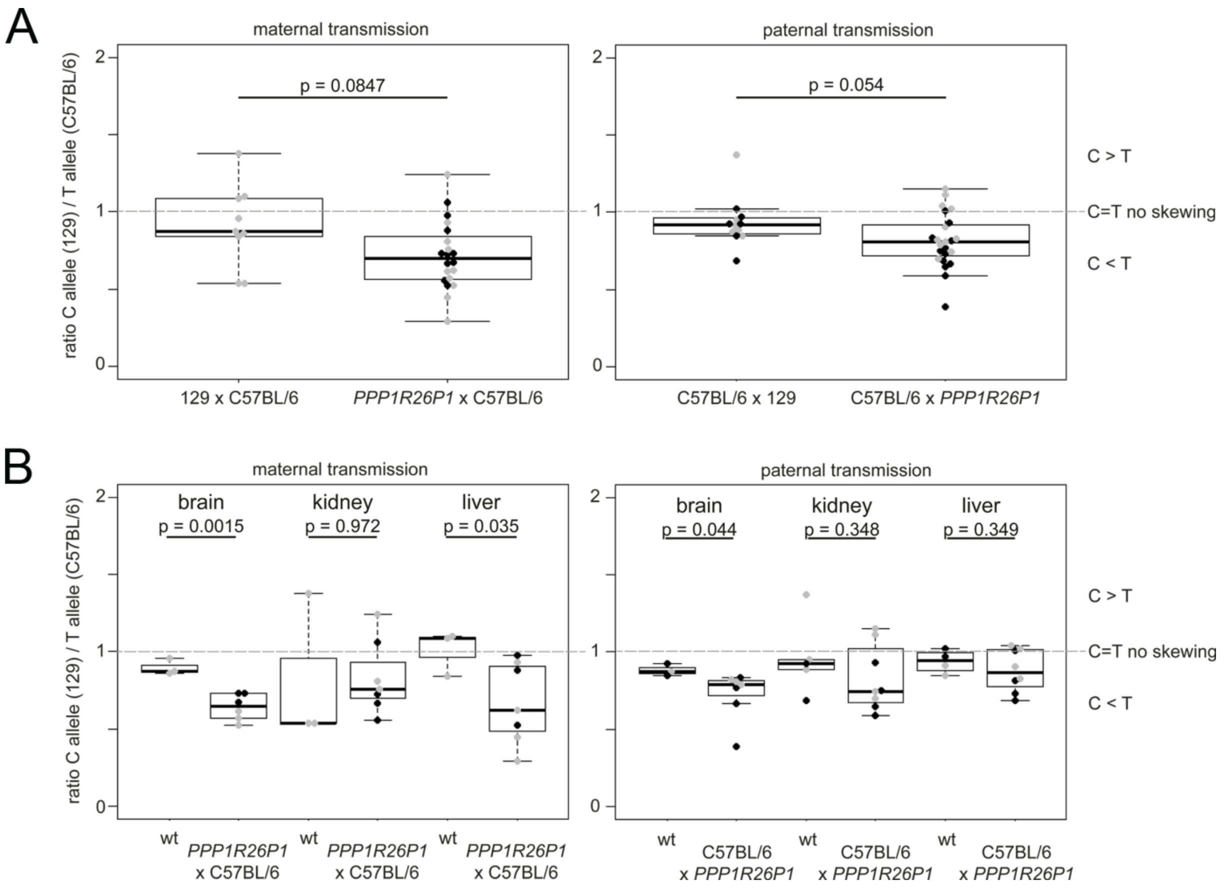

S3 Fig.: Detailed results of SNaPshot analyses. A) Level of allelic *Rb1* expression in heterozygous *Rb1_PPP1R26P1* mice either after maternal (left) or paternal (right) transmission of the pseudogene, compared to the respective wildtype control. Results of all tissues are included in one plot. B) Level of allelic *Rb1* expression depicted per tissue and per type of transmission of the pseudogene. Grey: female animals, black: male animals. P-values were calculated using Welch's t-test.
