# Supplementary material for: A Mouse Model for Imprinting of the Human Retinoblastoma Gene
Source: PLoS One. 2015 Aug 14;10(8):e0134672. doi: 10.1371/journal.pone.0134672 (PMC4537222; doi:10.1371/journal.pone.0134672)
Supplement: S1 Table — (PDF) [file pone.0134672.s005.pdf]

S1 Table: Result of karyotyping of eight correctly targeted ES cell clones.

Clone ID	Number of metaphase counted																			
	**1**	**2**	**3**	**4**	**5**	**6**	**7**	**8**	**9**	**10**	**11**	**12**	**13**	**14**	**15**	**16**	**17**	**18**	**19**	**20**
**23**	41	41	42	41	41	40	41	40	40	41	40	41	41	42	42					
**24**	40	40	40	40	40	40	40	40	40	40	40	40	40	40	40	40	40	40	40	40
**42**	40	40	40	40	40	40	40	40	40	40	40	40	40	40	40	40	40	40	40	40
**62**	39	39	39	39	39	39	39	39	39	39	39	39	39	39	39					
**71**	40	40	40	40	40	40	40	40	40	40	40	40	40	40	40	40	40	40	40	40
**75**	41	41	41	41	41	41	41	41	41	41	41	41	41	41	41					
**83**	40	40	40	40	40	40	40	40	40	40	40	40	40	40	40	40	40	40	40	40
**84**	41	41	41	41	41	41	41	41	41	41	41	41	41	41	41					
