# Supplementary material for: A Mouse Model for Imprinting of the Human Retinoblastoma Gene
Source: PLoS One. 2015 Aug 14;10(8):e0134672. doi: 10.1371/journal.pone.0134672 (PMC4537222; doi:10.1371/journal.pone.0134672)
Supplement: S2 Table — (PDF) [file pone.0134672.s006.pdf]

S2 Table: Chromosomal positions of analyzed regions

**name**	**UCSC browser assembly**	**position**
PPP1R26P1	GRch37/hg19	chr13:48,890,115-48,895,391
Rb1 intron 2	NCBI37/mm9	chr14:73,707,316-73,713,900
CpG146	NCBI37/mm9	chr14:73,724,977-73,726,383
CpG42	GRCh37/hg19	chr13:48,890,958-48,891,549
CpG85	GRCh37/hg19	chr13:48,892,636-48,893,857
AluSg	GRCh37/hg19	chr13:48,894,688-48,894,987
E2BAlu	GRCh37/hg19	chr13:48,894,312-48,894,594
