# Supplementary material for: A Mouse Model for Imprinting of the Human Retinoblastoma Gene
Source: PLoS One. 2015 Aug 14;10(8):e0134672. doi: 10.1371/journal.pone.0134672 (PMC4537222; doi:10.1371/journal.pone.0134672)
Supplement: S3 Table — CGI: CpG island analyzed, m, f: male or female; mat, pat: maternal or paternal inheritance of PPP1R26P1. Br: brain, li: liver, sp: spleen, ki: kidney, lu: lung, he: heart, sk: skin, mu: skeletal muscle. (PDF) [file pone.0134672.s007.pdf]

S3 Table: Summary of DNA methylation analyses in *PPP1R26P1* heterozygous mice.

CGI	animals	sex	transmission	tissues
**146**	2	male	1 mat, 1 pat	br, eye, li, blood, sperm
**42**	4	2 male<br>2 female	1 mat, 1 pat<br>1 mat, 1 pat	sp, ki, li, lu, he, sk, mu, br, eye, blood, sperm/ovary
**m*Rb1*_CpG85**	20	10 female<br>10 male	5 mat, 5 pat<br>5 mat, 5 pat	sp, ki, li, lu, he, sk, mu, br, eye, blood, sperm/ovary in four animals<br>br, ki, sperm/ovary in 16 animals
**E2BAlu**	2	male	1 mat, 1 pat	br, eye, li, blood, sperm
**m*Rb1*_AluSg**	2	male	1 mat, 1 pat	br, eye, li, blood, sperm

CGI: CpG island analyzed, m, f: male or female; mat, pat: maternal or paternal inheritance of *PPP1R26P1*. Br: brain, li: liver, sp: spleen, ki: kidney, lu: lung, he: heart, sk: skin, mu: skeletal muscle.
