# Supplementary material for: A Mouse Model for Imprinting of the Human Retinoblastoma Gene
Source: PLoS One. 2015 Aug 14;10(8):e0134672. doi: 10.1371/journal.pone.0134672 (PMC4537222; doi:10.1371/journal.pone.0134672)
Supplement: S4 Table — m, f: male or female; mat, pat: maternal or paternal transmission of PPP1R26P1; SPxx: identification numbers of mice. (PDF) [file pone.0134672.s008.pdf]

S4 Table: Results of DNA methylation analyses in percent

DNA methylation at the *Rb1* promoter, CpG146

**CpG146**	SP18 m, mat	SP30 m, pat								
brain	1.0	0.9								
eye	0.9	1.1								
liver	0.8	0.8								
blood	0.8	0.9								
sperm	0.6	0.5								

DNA methylation at CpG42

**CpG42**	SP18 m, mat	SP30 m, pat	SP23 f, mat	SP35 f, pat						
brain	46.3	52.2	49	43.3						
eye	55.1	56.6	53.1	54.3						
liver	6.0	4.7	1.8	2.9						
blood	1.5	2.3	1.8	2.4						
ovary / sperm	7.7	14.1	20.7	5.6						
heart	7.9	5.9	8	8.9						
lung	4.2	2.8	4.3	15.9						
muscle	15.8	21.8	35.9	26						
kidney	11.3	11.9	10.5	13.6						
spleen	2.6	3.5	4.7	5.9						
skin	1.5	2.3	1.8	2.4						

DNA methylation at CpG85, females

**CpG85**	SP23 f, mat	SP42 f, mat	SP43 f, mat	SP60 f, mat	SP66 f, mat	SP34 f, pat	SP35 f, pat	SP51 f, pat	SP56 f, pat	SP74 f, pat
brain	22.7	15.9	17.3	23.2	13.3	20.8	14	22.2	28.4	27.1
eye	25.2						29.3			
liver	0.9						0.8			
blood	1.4						2.4			
ovary	7.1	3.6	7.8	5.7	6.1	5	3	6.6	7.4	7.9
heart	3.5						1.4			
lung	0.3						8.1			
muscle	3.2						2.1			
kidney	7.6	8.8	4.8	3	4.3	7.4	7.7	7.4	3.6	2.8
spleen	3.3						3.4			
skin	0.6						0.8			

DNA methylation at CpG85, males

**CpG85**	SP18 m, mat	SP40 m, mat	SP41 m, mat	SP59 m, mat	SP63 m, mat	SP28 m, pat	SP29 m pat	SP30 m, pat	SP48 m, pat	SP69 m, pat
brain	21.5	18.9	24.7	24.9	14.7	26.3	17.5	14.1	23.7	22.1
eye	27.2							29.3		
liver	0.8							0.8		
blood	1.9							1.6		
sperm	3.0	3.3	2.5	2.9	2	2.5	1.1	0.8	2.1	1.9
heart	1.9							3.2		
lung	1.1							0.9		
muscle	2.4							2		
kidney	5.4	7.0	7.6	6.7	7.6	6.7	8.2	10.2	6	9
spleen	3.7							3.5		
skin	0.9							1.3		

DNA methylation at E2BAlu and AluSg

	E2BAlu		AluSg	
	SP18 m, mat	SP30 m, pat	SP18 m, mat	SP30 m, pat
brain	67.2	58.4	85.7	86.4
eye	56.9	67.7	63.7	65
liver	24.4	17.6	46.8	38.9
blood	40.8	43.9	74.1	61
sperm	16.7	29.1	40.4	39.5

DNA methylation in oocytes and sperm

**sperm**		**CpG14**	**CpG42**	**CpG8**	**E2BAl**	**AluSg**	***Snrpn***
SP18	mat	0.6	7.7	3	16.7	40.4	49.6
SP40	mat			3.3			61.4
SP41	mat			2.5			
SP59	mat			2.9			
SP63	mat			2			
SP220	mat		4.5	0.9		26.7	26.7
SP28	pat			2.5			58.6
SP29	pat			1.1			
SP30	pat	0.5	14.1	0.8	29.1	39.5	58.6
SP48	pat			2.1			
SP69	pat			1.9			
SP193	pat		2.6	1.7		18.2	7.5
SP132	mat/pat		0.7	1.1		1.9	0.9
SP141	mat/pat		2.2	1.2		15.7	14.0
**oocytes**							
SP233	mat			1.4		12.8	22.5
SP162	mat			0.9		25.6	83.4
SP232	mat			6.5		3.5	61.8
SP165	pat			0.4		9.1	43.3
SP99	pat			4.7		10.9	75.8
SP215	pat			3.4		14.1	47.7
SP207	mat/pat			0.5		0.4	1.5
SP237	mat/pat			3.6		12.5	93.9
SP228	mat/pat			0.3		0.3	0.4

DNA methylation at CpG85 in homozygous animals

**CpG85**	SP132 m	SP141 m	SP174 f	SP228 f	SP22 f
Blood	1.9	1.1	1.8	1.5	1.5
Brain	27	31.7	48.9	12.8	21.4
kidney	8.9	7	6.9	7	5.8
Sperm/ovary/o	1.1	1.2	4		

DNA methylation at *Snrpn* on chromosome 7C

	SP23 f, mat	SP35 f, pat	SP18 m, mat	SP30 m, pat
brain	50.2	53.4	48.3	67.0
ovary, sperm	54.5	46.6	49.6	28.0

m, f: male or female; mat, pat: maternal or paternal transmission of *PPP1R26P1*; SPxx: identification numbers of mice.
