# Supplementary material for: A Mouse Model for Imprinting of the Human Retinoblastoma Gene
Source: PLoS One. 2015 Aug 14;10(8):e0134672. doi: 10.1371/journal.pone.0134672 (PMC4537222; doi:10.1371/journal.pone.0134672)
Supplement: S5 Table — (PDF) [file pone.0134672.s009.pdf]

S5 Table: Primer sequences

Primer name	Sequence 5'->3'	Tm	Product size
**Gentoyping**			
*Rb1*_KIAA_KIAA_F	tcttgacctgaccaacgagc	60°C	210bp wildtype<br>362 bp *PPP1R26P1*
*Rb1*_KIAA_R_new	agctctctgcatgctccaat		
*Rb1*_KIAA_WT_F_new	gaggcaggagagtcacaagt		
neo_F	gcgcagctgtgctcgacgtt	60°C	522bp
neo_R	gaatcgggagcggcgataccg		
**Next generation bisulfite amplicon sequencing**			
m*Rb1*_CpG85_bis_out_fwd	ggtttgtagaaagggatttgttgt	56°C	549 bp
m*Rb1*_CpG85_bis_out_rev	ttcaaaaacaccacaaaataaaaac		
m*Rb1*_AluSg_bis_out_fwd	ttttatttggattggggagtaatt	56°C	386 bp
m*Rb1*_AluSg_bis_out_rev	cacccaaactaaaaaacaataaaac		
mSnrpn_Lucifero_outside_fwd_bis	tatgtaatatgatatagtttagaaattag	55°C	523 bp
mSnrpn_Lucifero_outside_rev_bis	aataaacccaaatctaaaatattttaatc		
CpG146_m*Rb1*_bisF1	cttgcttcctggcacgag-gtttttggagataagggaagatg	54°C	382 bp
CpG146_m*Rb1*_bisR1	caggaaacagctatgac-tcctctaccaaaaacctactatataac		
CpG42_KIAA_bisF1	cttgcttcctggcacgag-ggtttagatttttattgttgagttgt	54°C	304 bp
CpG42_KIAA_bisR1	caggaaacagctatgac-tctttaaaaacccacacttactaac		
Bis_m*Rb1*_CpG85_Ftag	cttgcttcctggcacgag-gggtagttttggaaatgtttaagatt	52°C	262 bp
Bis_m*Rb1*_CpG85_RM13	caggaaacagctatgac-aaaaaaacacccaactaaaaaaaac		
Bis_m*Rb1*_AluSg_Ftag	cttgcttcctggcacgag-atttttttgttagttttatatatttaaaag	50°C	235 bp
Bis_m*Rb1*_AluSg_RM13	caggaaacagctatgac-aaactacaaactcacaccaccac		
Bis_*RB1*_E2BAlu-Ftag	cttgcttcctggcacgag-ttagtaataagattataggtagg	46°C	318 bp
Bis_*RB1*_E2BAlu-RM13	caggaaacagctatgac-ctactccaaaaattactcc		
mSnrpn_Lucifero_inside_fwd_bis_Ftag	cttgcttcctggcacgag-aatttgtgtgatgtttgtaattatttgg	60°C	423 bp
mSnrpn_Lucifero_inside_rev_bis_Rtag	caggaaacagctatgac-ataaaatacactttcactactaaaatcc		
AutMID1 (example)	cgtatcgcctccctcgcgcca-tcag-acgagtgcgt-cttgcttcctggcacgag	59°C	+35 bp
ButMID1 (example)	ctatgcgccttgccagcccgc-tcag-acgagtgcgt-caggaaacagctatgac		
**RT-PCR, qRT-PCR**			
CpG85_fw	ctgcccttgttctcctgct	60°C	420 bp
*Rb1*_ex3_R	gcgataaagatgcagatgcccca		
CpG85_fw	ctgcccttgttctcctgct	60°C	593 bp
m*Rb1*_ex4_R	tgctgtagagtgcacataacacatt		
*Rb1*_ex2_F	acccgatcatgtcagagaaagagct	60°C	292 bp
m*Rb1*_ex4_R	tgctgtagagtgcacataacacatt		
transcript_2B_UPL_F	gtcctcactggatctcacctg	60°C	87 bp
transcript_2B_UPL_R	agatgccccagagttccttc		UPL probe 72
*Rb1*_ex4/6_UPL_F	aattagaacggacgtgtgaactt	60°C	95 bp
*Rb1*_ex4/6_UPL_R	ccaagaaatttttagcaccaaca		UPL probe 3
**SNaPshot**			
*Rb1*_gDNA_rs30444047_F	caagcatctccatggttcct	58°C	432 bp
*Rb1*_gDNA_rs13465574_R	tgggagaatcagctgcttgc		
*Rb1*_cDNA_rs304444047_F	tctctgcttttgcattcgtg	62°C	369 bp
*Rb1*_CDNA_rs304444047_R	ttcacccttacggattcctg		
*Rb1*_Snap_rs30444047_F	accatctggtttattttctggaactt	54°C	
